# Colonization during a key developmental window reveals microbiota-dependent shifts in growth and immunity during undernutrition

**DOI:** 10.1186/s40168-024-01783-3

**Published:** 2024-04-09

**Authors:** Yadeliz A. Serrano Matos, Jasmine Cano, Hamna Shafiq, Claire Williams, Julee Sunny, Carrie A. Cowardin

**Affiliations:** 1 Division of Pediatric Gastroenterology & Hepatology, Department of Pediatrics, University of Virginia School of Medicine, Charlottesville, VA 22908 USA; 2 Department of Microbiology, Immunology and Cancer Biology, University of Virginia School of Medicine, Charlottesville, VA 22908 USA

**Keywords:** Childhood undernutrition, Gastrointestinal microbiome, Linear growth; stunting, Gnotobiotic models, Infant microbiome, Mucosal immunity

## Abstract

**Background:**

Childhood undernutrition is a major global health challenge with devastating lifelong consequences. Linear growth stunting due to undernutrition has been linked to poor health outcomes, and mothers who experience growth stunting in childhood are more likely to give birth to stunted children later in life. Based on these findings, we hypothesized that intergenerational colonization of mice with microbiota from human donors with undernutrition may recapitulate certain immune and growth changes observed in this disorder.

**Results:**

To test this hypothesis, we developed a gnotobiotic murine model of undernutrition using microbiota from human infants with healthy or stunted growth trajectories. Intergenerational colonization with microbiota derived from children with growth stunting lead to less linear growth and the development of immune features of undernutrition and enteropathy, including intestinal villus blunting, lower liver IGF-1 and accumulation of intraepithelial lymphocytes and plasma cells in the small intestine. In contrast, colonization after weaning lead to fewer host phenotypic changes between these distinct microbial communities.

**Conclusions:**

These results are broadly consistent with previous findings demonstrating that exposure of the immune system to microbial products during the weaning phase is a critical determinant of later life immune function. Overall, our results suggest intergenerational colonization with human microbiota samples is a useful approach with which to investigate microbiota-dependent changes in growth and immunity in early life. Murine models that capture the intergenerational and multifactorial nature of undernutrition are critical to understanding the underlying biology of this disorder.

Video Abstract

**Supplementary Information:**

The online version contains supplementary material available at 10.1186/s40168-024-01783-3.

## Background

Childhood undernutrition is a formidable global health challenge, contributing to nearly half of all deaths in children under the age of five [1, 2]. The first 1,000 days of life, spanning from conception to age two, are widely recognized as critical in determining developmental outcomes, and undernutrition during this period can have devastating consequences [3, 4]. Linear growth stunting (length-for-age Z score ≤ 2 standard deviations below the WHO median) is a major feature of undernutrition impacting 149.2 million children globally in 2020 [1, 5]. Mothers who experience growth stunting as children are more likely to give birth to stunted children later in life, leading to a cycle of intergenerational transmission that has proven difficult to disrupt [6, 7]. Supporting this observation, among the best predictors of attained height in children are the child’s weight and length at birth and mother’s attained height, emphasizing the importance of development in utero and in early life [8–12]. The negative consequences of growth stunting persist into adulthood and include poor cognitive development, reduced educational attainment, and increased risk of metabolic and infectious disease [4, 13]. This syndrome is multifactorial and driven by inadequate nutrition, altered gut microbial communities, intestinal inflammation and pervasive pathogen colonization [14, 15]. These combined insults can drive a subclinical syndrome of intestinal epithelial derangement, inflammation, and barrier dysfunction known as Environmental Enteric Dysfunction (EED), which is prevalent in areas with high rates of undernutrition. EED is thought to limit the efficacy of therapeutic foods by decreasing the absorptive capacity of the small intestine [16–18]. Hallmark features of EED include epithelial remodeling and immune activation, with more small intestinal antibody-producing plasma cells, regulatory and cytotoxic T cells, and fewer intestinal macrophages [19–21]. Despite measurable progress in reducing stunting due to undernutrition, many of its long term consequences have proven resistant to pharmaceutical and nutritional therapies, highlighting the need to further understand the underlying etiology and mechanisms driving pathology [22, 23].

The gut microbiome plays a critical role in shaping both local and systemic immunity, and children with undernutrition are known to have altered gut microbial communities [24–26]. Transplantation of microbes from undernourished human donors to recipient germ-free (GF) animals suggests these community alterations are causally linked to deficits in growth and alterations in metabolism [26, 27]. Murine models have likewise highlighted early life as a critical window in which the immune system develops in reaction to the gastrointestinal microbiome [28, 29]. Indeed, GF mice that are colonized after the weaning period display heightened susceptibility to inflammatory pathologies [29]. Thus, the timing of colonization can dictate later immune outcomes, with significant implications for the long-term sequelae of undernutrition, many of which can be linked to immune dysfunction. These findings also raise the question of whether immunity differs depending on the composition of the microbiota that is present during critical periods of development.

To address these questions, we designed a murine model of early life undernutrition using human gut microbiota transmitted vertically from parents to offspring. In this “intergenerational” model, GF breeding mice were colonized with microbiota obtained from human infants with healthy or stunted growth trajectories. Offspring born to these mice inherited distinct microbial communities and were weaned onto a nutrient-deficient diet, capturing a critical window of early life development. We compared these mice to animals born GF but colonized directly after the weaning period. We demonstrate that intergenerational colonization with gut microbiota from human infant donors with linear growth stunting leads to less growth, small intestinal villous shortening, and significant intestinal immune alterations. These changes arise when animals are born to colonized parents, but not when colonized directly after weaning. We suggest that this model may serve as a useful tool with which to delineate the role of specific microbiota-dependent immune changes and their functional consequences during early life undernutrition.

## Methods

### Donor microbiota and study protocol

Microbiota donors for this study were infants born to women enrolled in the iLiNS-DYAD-M study. Details of enrollment for this study were described in an earlier publication [24]. Briefly, enrollment was open to consenting women over the age of 15 years with ultrasound confirmation of pregnancy of < 20 weeks gestation in the Mangochi District of southern Malawi. The randomized controlled clinical trial [clinicaltrials.gov #NCT01239693] tested the effects of providing small quantity Lipid-based Nutrient Supplements (SQ-LNS) to pregnant and lactating women through 6 months postpartum and to their children through 6–18 months of age [30]. During pregnancy and 6 months thereafter, women received one daily capsule of iron-folic acid supplement (IFA group), one capsule containing 18 micronutrients (MMN group), or one 20-g sachet of SQ-LNS [lipid-based nutrient supplements (LNS), containing 21 micronutrients, protein, carbohydrates, essential fatty acids, and 118 kcal]. Children in the IFA and MMN groups received no supplementation; children in the LNS group received SQ-LNSs from 6 to 18 months [31]. Donors used in this study were selected from samples collected at the six month time point (prior to supplementation) from a broader subset of donors based on their ability to colonize recipient gnotobiotic mice at an efficiency of > 50% (more than half of taxa present in the original donor sample were identified in initial experiments [24]). No significant differences in infant microbiota composition based on maternal supplementation at this time point were identified [32, 33].

### Gnotobiotic mice

All gnotobiotic mouse experiments were performed using protocols approved by the University of Virginia Institutional Animal Care and Use Committee. All gnotobiotic animals used in this publication were germ-free C57BL/6NTac mice obtained from Taconic Biosciences. Germ-free mice at Taconic receive autoclaved NIH-31 M diet and are housed in sterile flexible-film isolators. Upon arrival, germ-free status was verified by quantitative PCR. Mice were housed in plastic flexible film gnotobiotic isolators (Class Biologically Clean Ltd.) under a 12-h light cycle. Animals received ad libitum access to food and water throughout the experiment, and were euthanized at the conclusion of the experiment using AVMA approved procedures. For Post-Weaning experiments, male and female mice were obtained from Taconic Biosciences at 4 weeks of age, and were immediately transitioned to the Malawi-8 (M8) diet upon arrival. Three days later, they were colonized with donor microbiota and maintained thereafter on M8 until the time of euthanasia at 8 weeks of age. For Intergenerational experiments, male mice were obtained from Taconic Biosciences at 4 weeks of age and were immediately transitioned to the M8 diet upon arrival. Three days later, they were colonized with donor microbiota and maintained on M8 diet until 8 weeks of age. At this point, 8 week old germ-free females were introduced into the donor isolators. Males and females were co-housed and switched to an autoclaved nutrient-sufficient breeder chow (LabDiet 5021 Autoclavable Mouse Breeder Diet, LabDiet Inc.). Breeding animals were maintained on this diet thereafter. Offspring of these breeders (both HD and SD) were weaned at 21 days of life onto the M8 diet, and maintained on this diet until euthanasia at 8 weeks of age. Breeding mice were refreshed after six months, and offspring used in these experiments were derived from breeders from two separate rounds of colonization (total of 4 males and 6 females per breeding isolator). All data shown represent results from a minimum of two separate litters. Weight and tail length measurements were collected after euthanasia at 8 weeks of age. Tails were measured using a standard laboratory ruler from the base of the tail to the tip along a straight line. Fecal samples were collected at the indicated time points and immediately frozen. Samples were stored at -80 °C until use.

### Colonization and diets

To prepare infant fecal samples for colonization of germ-free mice, aliquots of each sample were removed from storage at -80 °C, weighed, and immediately transferred into anaerobic conditions (atmosphere of 75% N2, 20% CO2 and 5% H2; vinyl anaerobic chambers from Coy Laboratory Products). Samples were subsequently resuspended in pre-reduced PBS containing 0.05% L-Cysteine Hydrochloride at a concentration of 10 mg/mL. Samples were vortexed for one minute and allowed to clarify by gravity for 5 min. The supernatant was removed to a fresh anaerobic tube and combined with an equal volume of sterile, pre-reduced PBS containing 0.05% L-Cysteine HCL and 30% glycerol. Gavage mixtures were aliquoted into sterile 2 mL screw cap tubes (Axygen) and frozen at -80 °C until use. To colonize recipient mice, pools of gavage mixtures were sterilized externally with ionized hydrogen peroxide (STERAMIST System, TOMI Inc.) to prevent contamination with environmental microbes while preserving sample microbiota and passed into each isolator after appropriate exposure time (20 min). Animals were colonized via a single oral gavage with a 200 µl volume of gavage mixture.

The micro and macro-nutrient deficient Malawi-8 diet was obtained from Dyets, Inc. The components of the diet were selected based on micro and macro-nutrient components identified in the USDA Nutrient Database, although not specific to the donors used in this study [24, 34]. Ingredients (corn flour, mustard greens, onions, tomatoes, ground peanuts, red kidney beans, canned pumpkin and peeled bananas) were cooked and combined in an industrial mixer. Dry pellets of the M8 diet were extruded, vacuum-sealed, and double bagged prior to sterilization by irradiation (Steris Co). The nutritional content of the cooked and irradiated diet was assessed by N.P. Analytical Laboratories as described in Blanton et al. [24]. LabDiet 5021 was sterilized by autoclaving at 129 °C and 13.2 PSI for 15 min. Sterility of both diets was routinely assessed by culturing pellets in Brain Heart Infusion (BHI) broth (Millipore), Nutrient broth (Millipore), and Sabouraud-Dextrose (Millipore) broth for five days at 37 °C under aerobic conditions, and in BHI broth and Thioglycolate broth (Difco) supplemented with 0.05% L-Cysteine Hydrochloride (Sigma) under anaerobic conditions. After the five-day liquid culture, cultures of all diets were plated on BHI agar supplemented with sheep blood (Thermo Scientific). All diets were stored at -20 °C prior to use.

### Histopathology and anthropometry

At the time of euthanasia, a 1 cm section of the proximal ileum was dissected from each mouse and fixed in 10% neutral-buffered formalin overnight at room temperature before being transferred to 70% Ethanol. Tissue processing and H&E staining were performed by the University of Virginia’s Research Histology Core. Samples were paraffin embedded and sectioned before mounting. Slides were stained with hematoxylin & eosin prior to imaging at a 20 × magnification using an EVOS M7000 microscope. To assess the histopathological features of the ileum, the stained tissues were scored in a blinded manner by two independent observers. Resulting scores were then averaged. Scores were assigned using a scoring system based off published findings in human intestinal biopsies [35]. Scoring parameters consisted of three qualitative features: immune cell infiltration (0, no visible increase in tissue area; 1, increase in immune cells present in < 50% of tissue area; 2, increase in immune cells present in > 50% of tissue area), villous architecture (0, majority of villi are > 3 crypt lengths long; 1, majority of villi are < 3 but > 1 crypt length long, with abnormality; 2, majority of villi are absent or < 1 crypt length long, with abnormality) and enterocyte injury (0, majority of enterocytes show tall columnar morphology; 1, < 50% of enterocytes show low columnar, cuboidal, or flat morphology; 2, > 50% of enterocytes show low columnar, cuboidal, or flat morphology). Cumulative scores were calculated as the sum of the averaged score for all three parameters. ImageJ was used to obtain two quantitative parameters consisting of ileum villus height (μm) and ileum muscularis thickness (μm). Two measurements were obtained for each parameter and averaged.

To measure femur length, the femur and tibia were harvested from the right rear leg of each animal and cleaned of muscle and connective tissue. Femurs were fixed for ≥ 48 h in 70% ethanol. The femur was isolated via gentle disarticulation from the patella. Measurements were taken using digital calipers (Fisherbrand) at the longest points of the bone by a blinded observer.

### Microbial sequencing

DNA was prepared from fecal samples by bead beating (BioSpec Products) in a solution containing 500µL of extraction buffer [200 mM Tris HCL (pH 8.0), 200 mM NaCl, 20 mM EDTA], 210µL of 20% SDS, 500µL phenol:chloroform:isoamyl alcohol (pH 7.9, 25:24:1, Calbiochem), and 500µL of 0.1-mm diameter zirconia/silica beads. The aqueous phase was removed and DNA purified by PCR Purification Kit (Qiagen). Pure DNA was quantified by Qubit dsDNA BR assay (Invitrogen). DNA was normalized to a 2 ng/ul concentration and 15 ng total DNA was used as template for subsequent PCR reactions. Bacterial V4 16S rRNA gene amplicons were generated using barcoded primers 515F-806R [36]. PCR was performed with Invitrogen High Fidelity Platinum Taq using the manufacturer’s suggested cycling conditions. No-template controls were run with every sample plate to ensure there was no contamination of the barcoded primers or reagents. Amplicons were purified using Qiagen Qiaquick Purification Kit following the manufacturer’s protocol. To confirm the presence of targeted amplicons, PCR products were subjected to gel electrophoresis followed by quantification using the Qubit hsDNA Assay (Invitrogen). Barcoded amplicons were pooled to a concentration of 4 nM then sequenced using the Miseq Reagent Kit v3 and Miseq platform (Illumina) following the manufacturer’s recommended protocols.

### Sequencing quality control & data analysis

Sequence reads were demultiplexed using Illumina Miseq Reporter Software. Quality checks were performed using fastqc version 0.11.5 before and after trimming. Samples with less than 5,000 reads were excluded from downstream analysis. Remaining reads were trimmed using the Bbduk tool from BBMap (https://sourceforge.net/projects/bbmap/) to 150 base pairs (bp) and with sequence quality qc ≥ 10. ASVs were generated using the DADA2 pipeline (version 1.26.0) in R (version 4.2.3). Taxonomic assignments were performed using the SILVA ribosomal RNA gene database (v138.1). ASVs were filtered based on abundance (> 0.1% across all samples) and prevalence (> 2% across all samples [37]). Ordination plots showing UniFrac distances were produced using the phyloseq package (version 1.42.0) in R [38]. The heatmap shown in Fig. 2A was created using the pheatmap package in R (version 1.0.12) with row normalization using the ‘scale’ function to center and scale the data. Stacked bar plots were generated using the miaViz package (version 1.9.0).

### Flow cytometry

#### Tissue harvest and cell isolation

The entire small intestine was dissected from each mouse and placed onto a moist piece of gauze soaked in HBSS with 10 mM HEPES (Gibco) to prevent drying. The intestine was separated into duodenum, jejunum, and ileum, and a small portion (~ 1 cm) of the proximal end of each section was removed for histological analysis. Intestinal contents were then gently squeezed out of each section. Peyer’s Patches and any fat were removed from the small intestine exterior, then the tissue was sliced lengthwise to expose the lumen, and samples were placed in 10 ml of HBSS with 10 mM HEPES on ice. Intestines were then transferred to 20 ml of room temperature epithelial removal buffer (1 × HBSS with 20% FBS (Gibco), 7.5 mM HEPES (Invitrogen), and 2.5 mM EDTA (Invitrogen)) and rotated on a tube rotator for 20 min at room temperature, then vortexed for 20 s. Intestines and buffer were then poured over a 100 µm filter and the collected cell suspension containing intestinal epithelial cells was spun at 500xg for 5 min at 4 °C, resuspended in 5 ml FACS buffer (1 × DPBS with 2% FBS), and reserved for flow cytometric staining and analysis. This process was repeated once more with each intestine sample and the remaining intact tissue at this point consisted of small intestine lamina propria. Tissue was then rinsed in 1 × HBSS with 10 mM HEPES to remove residual epithelial removal buffer, and finely chopped with scissors, then resuspended in 10 ml of warmed complete RPMI (Gibco) and 100ul of 100 U/mL collagenase IV (Millipore Sigma). Samples were incubated at 37 °C on a shaker at 180 rpm for 45 min. The resulting mixture was poured over a 40 µm filter and tissue mashed through the filter with the end of a syringe plunger. Plunger, filter, and original digestion tube were rinsed with up to 5 ml of FACS buffer to ensure maximum cell yield. Resulting cell suspension was spun at 500xg for 5 min at 4 °C, then the supernatant was decanted, and the cell pellet was resuspended in 4mls of 40% Percoll (Cytiva) in HBSS in a 15 ml conical tube. This Percoll layer was gently underlaid with 3 ml of 70% Percoll in HBSS to create two distinct layers. Tubes were spun at 850xg for 20 min at 4 °C with the centrifuge brake turned off to prevent mixing of the Percoll layers. After spin, the interface of cells between the two Percoll layers was collected and added to 10 ml of HBSS with 10 mM HEPES. Tubes were spun at 500xg for 5 min at 4 °C, then supernatant was aspirated off and cell pellets were resuspended in 500ul of FACS buffer.

#### Surface and intracellular staining for flow cytometry

Epithelium and lamina propria cell suspensions were plated in 96 well round bottom plates, spun at 500xg at 4 °C for 5 min, supernatant decanted, and 50ul of surface stain mixture (fluorescent antibodies for surface markers, TruStain FcX PLUS [Biolegend], and Zombie Aqua Fixable Viability dye [Biolegend]) in FACS buffer was applied to each well and incubated for 20 min at room temperature in the dark. Plates were then spun at 500xg at 4 °C for 5 min, and supernatant decanted. If cells were receiving no intracellular stains, cells were resuspended in 100ul of 1 × Fixation Buffer (BD Biosciences) for 20 min in the dark at 4 °C. They were then spun at 500xg at 4 °C for 5 min, supernatant was decanted, and they were resuspended in 200ul of FACS buffer and allowed to sit at 4 °C overnight in the dark. On the day of analysis, cells were spun at 500xg at 4 °C for 5 min, supernatant decanted, and resuspended in 200ul FACS buffer plus 5ul of CountBright Absolute Counting Beads (Thermo Fisher). For intracellular staining, cells were fixed in 100ul FoxP3 Fix/Perm Buffer (Invitrogen) for 20 min at 4 °C in the dark, then spun at 500xg at 4 °C for 5 min, supernatant was decanted, and cells were resuspended in 100ul of intracellular antibodies diluted in 1 × permeabilization buffer (Invitrogen) with 2% rat serum (Invitrogen) and incubated overnight at 4 °C in the dark. On the day of analysis, cells were spun at 500xg at 4 °C for 5 min, supernatant decanted, resuspended in 200ul of 1 × permeabilization buffer, and allowed to sit at room temperature for 5 min. Cells were then spun again, decanted, and resuspended in 200ul FACS buffer plus 5ul of CountBright Absolute Counting Beads (Thermo Fisher).

#### Analysis and gating

Samples were run on the Attune NxT Acoustic Focusing Cytometer with CytKick Auto Sampler and analyzed using FlowJo software. All cell types were first gated as live, single cells. Subsequent gating was performed as follows:

**Table:** 

**Tissue Type**	**Cell Type**		**Markers**	
Epithelium	αβ T Cells		CD45 + EpCAM- TCRβ +	
Epithelium	CD4 + T Cells		CD45 + EpCAM- TCRβ + CD4 +	
Epithelium	CD8 + T cells		CD45 + EpCAM- TCRβ + CD8 +	
Epithelium	γδ T Cells		CD45 + EpCAM- TCRγδ +	
Epithelium	CD8αα + TCRαβ Natural IEL		CD45 + EpCAM- TCRβ + CD8α + CD8β-	
Epithelium	CD8αα + TCRγδ Natural IEL		CD45 + EpCAM- TCRγδ + CD4- CD8β- CD8α +	
Epithelium	CD8αβ + TCRαβ Induced IEL		CD45 + EpCAM- TCRβ + CD8α + CD8β +	
Lamina Propria/Epithelium	Eosinophils		CD45 + CD11b + Ly6G- SiglecF + SSC high	
Lamina Propria/Epithelium	Monocytes		CD45 + CD11b + Ly6C + Ly6G-	
Lamina Propria/Epithelium	Neutrophils		CD45 + CD11b + Ly6C ± Ly6G +	
Lamina Propria/Epithelium	Macrophages		CD45 + CD11b + F4/80 + SiglecF-	
Lamina Propria	αβ T Cells		TCRβ +	
Lamina Propria	γδ T Cells		TCRγδ +	
Lamina Propria	CD4 + T Cells		TCRβ + CD4 + CD8-	
Lamina Propria	CD8 + T cells		TCRβ + CD4- CD8 +	
Lamina Propria	Rorγt +		TCRβ + CD4 + CD8- Rorγt +	
Lamina Propria	Treg		TCRβ + CD4 + CD8- FoxP3 +	
Lamina Propria	Rorγt + Treg		TCRβ + CD4 + CD8- FoxP3 + Rorγt +	
Lamina Propria	CD44 + T cells		TCRβ + CD44 +	
Lamina Propria	B Cells		B220 + CD19 +	
Lamina Propria	IgA + Plasma Cells		Lin- (CD3-TCRβ-CD4-CD11c-NK1.1-F4/80-) B220- IgA +	
**Target**	**Fluorophore**	**Clone**	**Vendor**	**Catalog#**
B220	PE-Cy7	RA3-6B2	Biolegend	103222
CD11b	PerCP-Cy5.5	M1/70	Biolegend	101228
CD11c	Brilliant Violet 421	N418	Biolegend	117330
CD19	Brilliant Violet 421	6D5	Biolegend	115538
CD4	FITC	RM4-5	Biolegend	100510
CD4	APC	RM4-5	Biolegend	100516
CD44	APC-Cy7	IM7	Biolegend	10,028
CD45	APC eFluor780	30-F11	eBioscience	47–0451-82
CD8α	PE-Cy7	53–6.7	Biolegend	100722
CD8β	FITC	YTS156.7.7	Biolegend	126605
EpCAM	Brilliant Violet 421	G8.8	Biolegend	118225
F4/80	APC	BM8	Biolegend	123116
FoxP3	APC	FJK-16 s	eBioscience	17–5773-82
Granzyme A	PE	3G8.5	Biolegend	149704
IgA	PE	mA-6E1	eBioscience	12–4204-83
LY6C	FITC	AL-21	BD Biosciences	553104
LY6G	PE-Cy7	1A8	Biolegend	127618
PD1	APC-Cy7	29F.1A12	Biolegend	135224
Rorγt	PE	B2D	eBioscience	12–6981-82
SiglecF	PE	E50-2440	BD Biosciences	552128
TCRβ	PerCP-Cy5.5	H57-597	Biolegend	109228
TCRγδ	Brilliant Violet 421	GL3	Biolegend	118120
TCRγδ	PE	GL3	Biolegend	118108
CD3	PerCP-Cy5.5	145-2C11	Biolegend	1000328
CD4	PerCP-Cy5.5	RM4-5	Biolegend	100539
CD11c	PerCP-Cy5.5	N418	Biolegend	117327
NK1.1	PerCP-Cy5.5	PK136	Biolegend	108728
F4/80	PerCP-Cy5.5	BM8	Biolegend	123127
Live dead	Aqua	–	Biolegend	423101

### Protein quantification

Liver and ileum tissue were collected at the time of euthanasia and flash frozen in liquid nitrogen. Liver and ileum samples were homogenized in Lysing Matrix F (MP Biomedicals, ref. 6540440) and 500uL of extraction buffer (1 × HALT (Thermo Scientific, ref. 78429) in T-PER (Thermo Scientific, ref. 78510)). Homogenates were centrifuged at 4 °C and 10,000 xg for 5 min. Protein concentration was determined by BCA assay (Thermo Scientific, ref. 23227) using the manufacturer’s protocol. Lysates were normalized to a total protein concentration of 1 mg/mL in extraction buffer and stored at -80 °C until analysis. A subset of ileum samples (randomly selected to include equal numbers of male and female mice from at least two separate litters) were sent to UVA’s Flow Cytometry Core Facility where the Luminex assay (MILLIPLEX MAP Mouse Cytokine/Chemokine 32-Plex Magnetic Bead Panel) was performed according to the manufacturer’s protocol, which is as follows: 200uL of Wash Buffer (room-temperature 1 × Wash Buffer in deionized water) was added to each well of a clean plate and sealed. Plate was mixed for 10 min on a plate shaker at 20–25 °C. After removing the wash buffer via magnetic plate washer, 25uL of standard or control was added to the corresponding wells. 25uL of assay buffer were added to background and sample wells. 25uL of previously mentioned lysis buffer was added to the background, standards, and control wells. After vortexing the mixing bottle, 25uL of the mixed or premixed beads were added to each well. The plate was sealed, covered with foil, and incubated overnight at 2–8 °C with agitation. After removing the well contents and washing the plate twice, 25uL of detection antibody was added to each well. The plate was sealed, covered with foil, and incubated for 1 h at 20–25 °C with agitation. 25uL of Streptavidin–Phycoerythrin was added to the wells containing detection antibody. The plate was sealed, covered with foil, and incubated for 30 min at 20–25 °C with agitation. After removing the well contents and washing the plate twice, 150uL of Sheath Fluid PLUS was added to all wells. The beads were resuspended on a plate shaker for 5 min and the plate was measured on the Luminex xMAP Intelliflex. Sample analyte concentration was calculated by fitting the median fluorescence intensity (MFI) data to that of a standard curve, validated by lot-matched quality controls using the Milliplex Analyst software.

CCL5 and IL-1β in ileum lysates and IGF-1 in liver lysates were quantified by ELISA assay (R&D Systems DuoSet kits) performed according to the manufacturer’s recommendation. Briefly, the capture antibody was coated onto a 96-well half-area plate (Corning) in PBS overnight at room temperature. On the following day, the plates were washed three times with 200 uL of Wash Buffer (0.05% Tween 20 in PBS) and blocked for one hour. After blocking and incubation, plates were washed again before adding 50 µl of tissue lysate as well as a standard curve. The plate was incubated for 2 h at room temperature before being washed as described above. Detection Antibody provided in the kit was added at the suggested concentration and incubated for another 2 h at room temperature. After incubation, plates were washed and Substrate Solution (1:1 mixture of Color Reagent A (H_2_O_2_) and Color Reagent B (Tetramethylbenzidine)) was added to each well and incubated at room temperature for 20 min avoiding direct light. After incubation, Stop Solution (2 N H_2_SO_4_) was added to each well. Plates were read at an optical density of 450 nm with background subtraction at 570 nm using a Tecan plate reader.

### Statistical analysis

Statistical analyses were performed in GraphPad Prism (version 9.5.1) unless otherwise noted. V4 16 s sequencing data was analyzed in R (version 4.2.3). Statistical details including the number of animals or samples can be found in figure legends. Statistical significance was assessed by Mann–Whitney U test when comparing between two groups, or by Two-Way ANOVA with Šídák’s multiple comparisons test when comparing > 2 groups unless otherwise noted. *P* values are shown in figures or tables for samples with significant differences. Each data point represents an individual animal and horizontal bars represent the mean.

## Results

### Establishing an intergenerational model of undernutrition

**Fig. 1:**
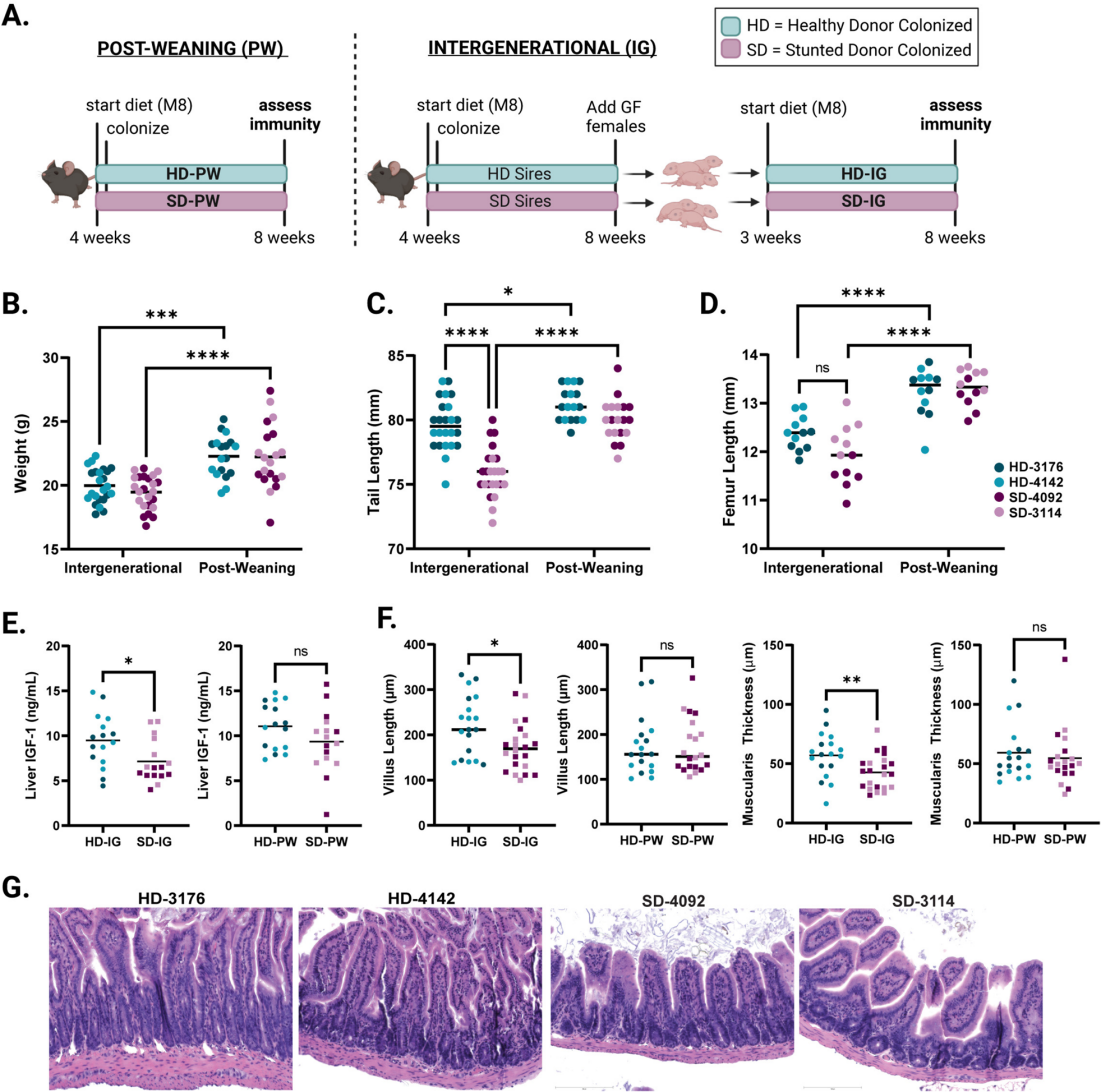
Development of a murine model of intergenerational undernutrition. **A** Schematic of experimental design (created with Biorender.com). Both HD and SD pups in the PW model are maintained on the M8 diet from 4–8 weeks of age. HD and SD pups in the IG model are likewise weaned onto the M8 diet at 3 weeks of age. **B** Absolute weight of animals at the time of euthanasia**. C** Tail length. **D** Femur length. **E** Measurement of IGF-1 in liver tissue of IG and PW mice. **(F)** Representative histological images of H&E stained ileal tissue from IG mice. **G** Quantification of villus length and muscularis thickness in IG and PW mice. Data shown pooled between healthy donor colonized mice (3176 and 4142) and stunted donor colonized mice (4092 and 3114) at 8 weeks of age. Each point represents an individual animal. **B-D** * *p* ≤ 0.05, **** *p* ≤ 0.0001 by Two-Way ANOVA with Šídák’s multiple comparisons test. **B-C** *n* = 24/group [12 per donor] for IG groups, *n* = 18-20/group [8-10 per donor] for PW groups. **D** *n* = 12/group [6 per donor] for all groups in all conditions (**E**) *n* = 16/group [8 per donor] for all groups. **F-G** *n* = 18-22/group [7-12 per donor] for IG groups, *n* = 18-20/group [8-10 per donor] for PW groups. **E-G** * *p* ≤ 0.05, ** *p* ≤ 0.01 by Mann-Whitney U test

In order to determine whether immune developmental outcomes would differ depending on the composition of the gut microbiota during early life, we colonized young (4-week-old) GF C57Bl/6 mice with fecal microbiota sampled from one of four donors: two healthy (height-for-age Z score (HAZ) = 1.12 and 1.74) or two stunted (HAZ = -3.35 and -2.33) six-month-old Malawian infants (Fig. S1A) [24, 30]. Mice were fed a micro and macro-nutrient deficient diet composed of foods commonly consumed in Malawi (Malawi-8 or M8 [24]). In one arm of the experiment, male and female GF mice were colonized at 4 weeks of age and maintained on the M8 diet until 8 weeks, when microbiota composition and immune phenotypes were assessed. These animals represent the “Post-Weaning” (PW) model of colonization (Fig. 1A). In a second arm of the experiment, male mice were similarly colonized and maintained on M8 from 4 to 8 weeks of age. At 8 weeks, age-matched GF females were introduced, breeding pairs were cohoused and maintained thereafter on a nutrient-sufficient diet. Male and female offspring from these breeding pairs were weaned onto the M8 diet from 3 to 8 weeks of age. These animals represent the “Intergenerational” (IG) model of colonization (Fig. 1A). To identify phenotypes that differed based on donor growth status, we assessed results by combining data from both stunted donors (SD) and comparing against combined data from both healthy donors (HD).

### Intergenerational colonization with SD microbiota leads to less linear growth

We next assessed growth phenotypes in both IG and PW animals at 8 weeks of age. We found no difference in overall weight between HD and SD animals colonized either intergenerationally or post-weaning (Fig. 1B); however, both groups weighed significantly more when colonized after weaning, possibly due to an additional week of undernutrition in the IG groups. Consistent with these results, we found no significant differences in weight between groups of IG dams and sires after colonization, or between SD-PW and HD-PW mice in the first two weeks after colonization **(**Fig. S1B,C). In contrast, we noted significantly lower tail length, a surrogate for linear growth, in SD-IG relative to HD-IG mice (Fig. 1C). No difference in tail length was detected between SD-PW and HD-PW groups. Overall, both IG groups had significantly shorter tails than their PW counterparts, consistent with the finding of lower body weight. As a second measure of linear growth, we also assessed femur length, which showed similar outcomes, although differences between SD-IG and HD-IG groups did not reach statistical significance (Fig. 1D). Interestingly, femur length differences appeared more severe in SD-4092 animals compared to the SD-3114 animals, suggesting differences in community composition between the two stunted donors are likely involved. These trends were evident when comparing between IG and PW mice in each individual donor group and were not sex-dependent (Fig. S1D-H). In human cohorts and other mouse models of undernutrition, levels of Insulin-like Growth Factor-1 (IGF-1) correlate strongly with linear growth [39, 40]. We next assessed levels of IGF-1 in the liver by ELISA, and found significantly lower IGF-1 in SD-IG animals relative to the HD-IG group (Fig. 1E), with no significant difference identified between SD-PW and HD-PW mice. Over the course of the experiment, we did not find significant differences in litter size between IG groups colonized with distinct donor microbiota (Fig. S2A). Because all four IG groups showed less growth compared to PW animals, these differences could reflect paternal or early life M8 diet exposure or differences in pre-weaning diet in addition to earlier microbial colonization. These findings also suggest that intergenerational but not post-weaning colonization with SD microbiota negatively influences linear growth and liver IGF-1 relative to HD microbial communities.

### SD microbial communities influence intestinal histopathology

To begin to characterize underlying differences between SD-IG and HD-IG offspring that could explain the observed differences in linear growth, we next examined small intestine histopathology. Blinded scoring of hematoxylin and eosin-stained ileal tissue sections from all four donor groups revealed significantly lower quantitative measurements of villus length and muscularis thickness in SD-IG relative to HD-IG mice (Fig. 1F-G). Interestingly, these changes were not present when comparing HD-PW to SD-PW groups, suggesting that shorter villi were not a general consequence of the presence of specific microbes, but depended on when these microbes were encountered. In addition to these quantitative measures, we also subjected sections to blinded scoring using three subjective parameters commonly observed in human intestinal biopsies from patients with undernutrition and EED [17, 35, 41], including immune cell infiltration, villous architecture and enterocyte injury. Surprisingly, we did not identify significant differences in these parameters, which may depend on additional environmental or pathogen exposures not replicated in our model (Fig. S2B, Table S1A-B).

### Distinct microbial communities colonize all four donor groups

**Fig. 2:**
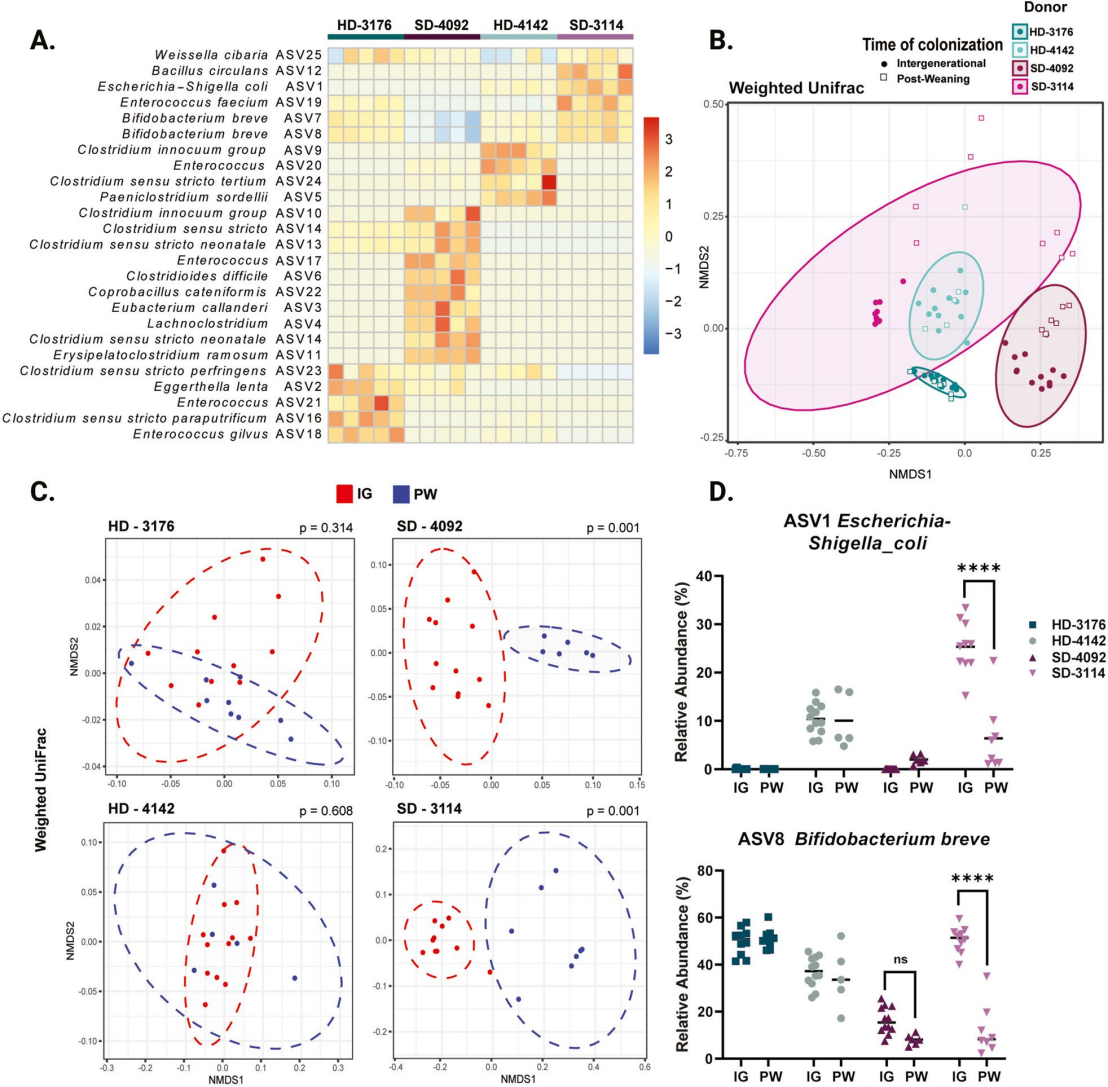
Microbial communities of recipient mice show distinct patterns of colonization. **A** Row-normalized heatmap of the top 25 ASVs by relative abundance for all four IG donor groups in fecal samples at 8 weeks of age. *n* = 5/group. **B** NMDS plot of weighted UniFrac distances of IG and PW fecal samples at 8 weeks of age by donor colonization. *n* = 5–12/group. **C** NMDS plot of weighted UniFrac distances of IG versus PW fecal samples at 8 weeks of age by individual donor. *n* = 5–11 mice/group with p value calculated by PERMANOVA. **D** Relative abundances of ASV1 *Escherichia-Shigella coli* and ASV 8 *Bifidobacterium breve* for all four IG and PW donor-colonized groups at 8 weeks of age. *n* = 5–12/group, * *p* ≤ 0.05, ** *p* ≤ 0.01, **** *p* ≤ 0.0001 for comparisons between IG and PW samples within each donor by Two-Way ANOVA with Šídák’s multiple comparisons test

In order to define the microbial communities mediating these effects, we next performed V4 16 s rRNA sequencing of the fecal microbiota from mice in all four donor groups. Analysis of samples from IG animals at maturity revealed distinct community configurations for each donor (Fig. 2A, S2C, Table S2A-E), with a modest number of shared taxa (Fig. S2D). Analysis of UniFrac distances demonstrated that the composition of the fecal microbiota was similar between PW and IG samples within each individual donor, with the greatest variability present in donor 3114, and to a lesser extent, donor 4092 (Fig. 2B, S2E). Direct comparisons of PW to IG samples for each donor by weighted (Fig. 2C) and unweighted (Fig. S2F) UniFrac demonstrated significant differences in community structure between PW and IG mice colonized with the two stunted donor communities (Table S2A-D). Similarly, Shannon Diversity was not significantly different between HD IG and PW groups but was significantly increased in SD PW versus IG groups (Table S2E). Because PW mice were colonized directly while IG mice received vertically transmitted microbes, these results suggest the HD communities may be more efficiently passed from parents to offspring. In contrast, SD microbiota appeared to be surprisingly dependent on the mode of colonization (Fig. 2D). Whether these divergent patterns represent poor intergenerational transmission by the SD communities, more efficient engraftment by direct oral gavage, or shaping of the community by differential host immune responses warrants further investigation.

### Intergenerational colonization shapes small intestinal intraepithelial lymphocytes

**Fig. 3:**
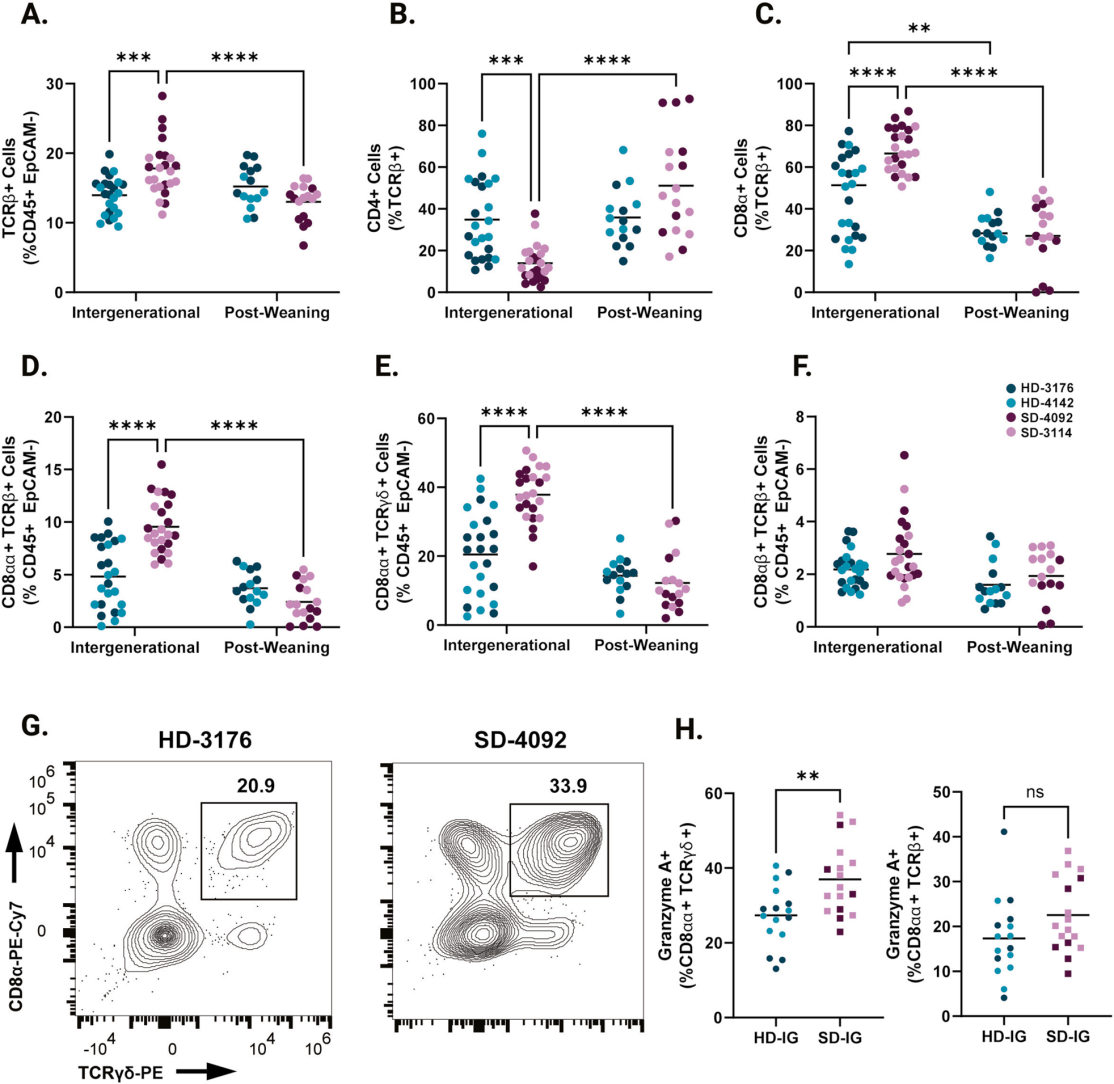
Immune cell composition of the small intestinal epithelium in IG and PW colonized mice at 8 weeks of age. **A** TCRβ + cells shown as a percentage of CD45 + EpCAM- Live cells. **B** CD4 + cells shown as a percentage of TCRβ + CD45 + EpCAM- Live cells. **C** CD8 + cells shown as a percentage of TCRβ + CD45 + EpCAM- Live cells. **D** CD8αα + TCRαβ Natural IELS (gated as CD8α + CD8β- TCRβ + cells as a percentage of CD45 + EpCAM- Live). **E** CD8αα + TCRγδ Natural IELs (gated as CD8α + CD8β-CD4- TCRγδ + cells as a percentage of CD45 + EpCAM- Live). **F** CD8αβ + TCRαβ Induced IELs (gated as CD8α + CD8β + TCRβ + cells as a percentage of CD45 + EpCAM- Live). **G** Representative flow plots of TCRγδ + Natural IELs in HD and SD IG mice. Cells were gated on the Live CD45 + EpCAM- population. **H** Granzyme A + cells shown as a percentage of CD8α + CD8β- CD4- TCRγδ + CD45 + EpCAM- Live cells (left) or CD8α + CD8β- TCRβ + CD45 + EpCAM- Live cells (right) within IG colonized mice. Data shown pooled between healthy donor colonized mice (3176 and 4142) and stunted donor colonized mice (4092 and 3114). Each point represents an individual animal. **A**-**F** *n* = 24/group [12 per donor] for IG groups, *n* = 18–20/group [7–10 per donor] for PW groups. * *p* ≤ 0.05, ** *p* ≤ 0.01, *** *p* ≤ 0.001, **** *p* ≤ 0.0001 by Two-Way ANOVA with Šídák’s multiple comparisons test. **H** *n* = 16–17/group [6–11 per donor], ** *p* ≤ 0.01 by Mann–Whitney U test

Based on differences in linear growth and intestinal histopathology between SD-IG and HD-IG mice, we next sought to determine whether these microbial communities influenced immune cell composition in the small intestine epithelium. We first identified significantly elevated TCRβ + cells in SD-IG relative to HD-IG mice (Fig. 3A, S3A and Table S3A-D), a difference that was not observed in HD-PW and SD-PW groups. Within this compartment, SD-IG mice had a significantly greater proportion of CD8α + TCRβ + cells and a significantly lower proportion of CD4 + TCRβ + T cells in the epithelium, whereas SD-PW and HD-PW mice did not (Fig. 3B-C, S3B-C). These results pointed towards potential differences in populations of intraepithelial lymphocytes (IELs), a heterogeneous group of microbiota-responsive immune cells located within the intestinal epithelium with diverse regulatory and inflammatory functions [42–45].

IELs exist as two major subsets, including ‘natural’ IELs that are activated within the thymus, and ‘induced’ IELs that derive from conventional T cells activated peripherally [43]. Intriguingly, SD-IG mice showed a significant increase in two subsets of natural IELs. CD8αα + TCRβ + and CD8αα + TCRγδ + IELs were both significantly more abundant in SD-IG mice relative to HD-IG animals, but neither cell type differed between the PW groups (Fig. 3D-E, G, and Fig. S3D-E). In contrast, induced CD8αβ + TCRβ + IELs did not significantly differ between groups (Fig. 3F, S3F). A significantly greater proportion of CD8αα + TCRγδ + IELs in SD-IG mice were also positive for Granzyme A, a cytotoxic mediator found in T lymphocytes (Fig. 3H) [21]. Based on these results, we concluded that differences in the proportions and function of natural IEL subsets were influenced by the composition of the microbiota when colonized intergenerationally, but not when colonized after weaning.

### *Rorγt* + *T regulatory cells are increased in SD-IG mice*

**Fig. 4:**
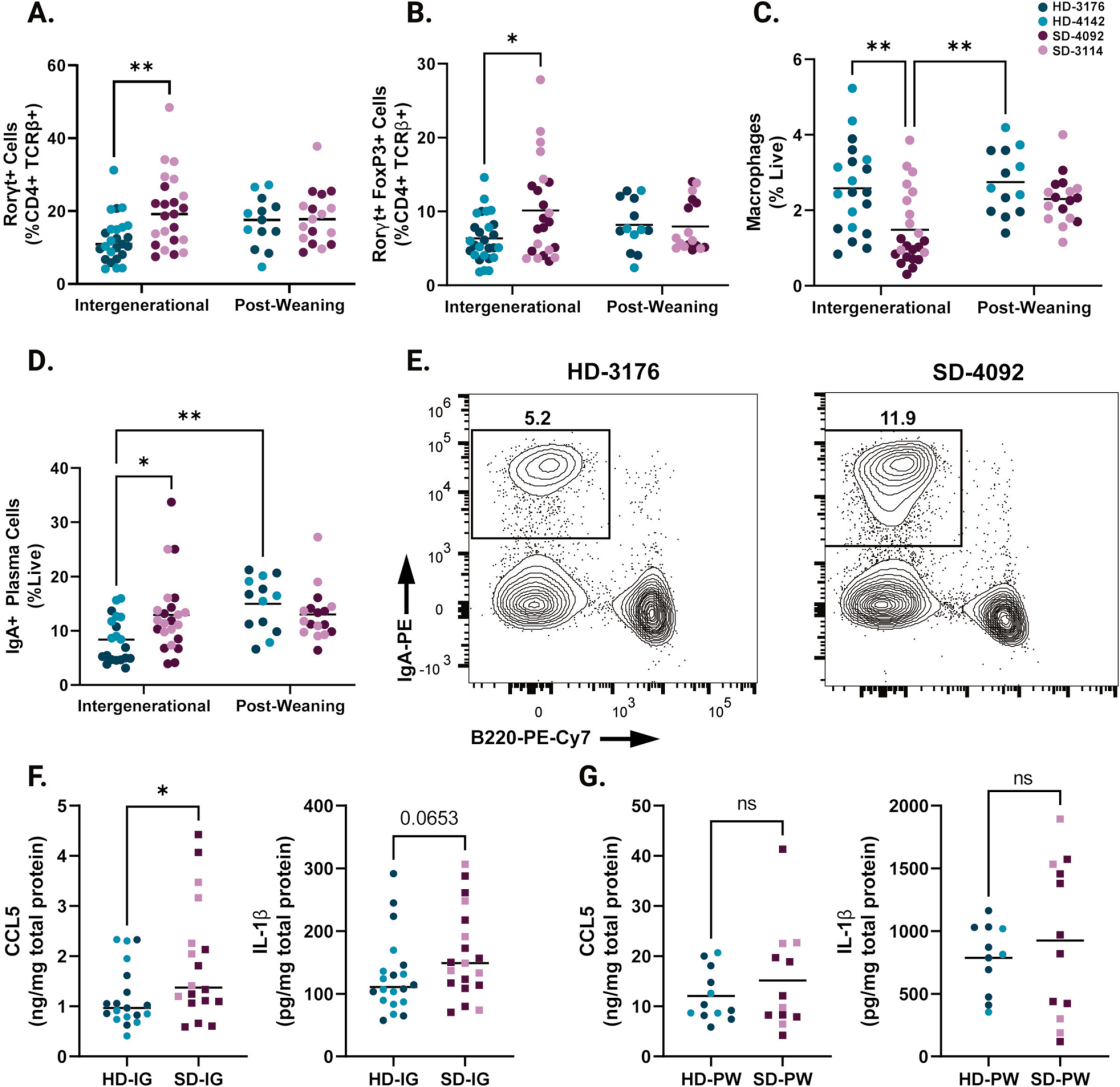
Immune features of the small intestine lamina propria in IG and PW colonized mice at 8 weeks of age. **A** Rorγt + cells in the small intestine lamina propria shown as a percentage of CD4 + TCRβ + live cells. **B** Rorγt + Regulatory T cells (gated as Rorγt + FoxP3 + CD4 + TCRβ + live cells) in the small intestine lamina propria shown as a percentage of CD4 + TCRβ + live cells. **C** Macrophages (gated as F4/80 + CD11b + CD45 + live cells) in the small intestine lamina propria shown as a percentage of live cells. **D** IgA + Plasma Cells (gated as Lin- [CD3-TCRβ-CD4-CD11c-NK1.1-F4/80-] IgA + B220- live cells) in the small intestine lamina propria shown as a percentage of live cells. **E** Representative flow plots of IgA + Plasma Cells (gated on live Lin- cells) in HD and SD IG groups. **F** Levels of CCL5 and IL-1β in ileal tissue lysates from IG mice as measured by ELISA. **G** Levels of CCL5 and IL-1β in ileal tissue lysates from PW mice as measured by ELISA. Data shown pooled between healthy donor colonized mice (3176 and 4142) and stunted donor colonized mice (4092 and 3114). Each point represents an individual animal. **A-D** *n* = 20–24/group [10–12 per donor] for IG groups, *n* = 14–17/group [5–9 per donor] for PW groups. * *p* ≤ 0.05, ** *p* ≤ 0.01, *** *p* ≤ 0.001, **** *p* ≤ 0.0001 by Two-Way ANOVA with Šídák’s multiple comparisons test. **F** *n* = 18–20/group [6–13 per donor], * *p* ≤ 0.05 by Mann–Whitney U test

We next investigated changes to innate and adaptive immune populations within the small intestine lamina propria. Overall, cellular changes in this compartment were less marked than those in the epithelium. However, we did note an overall increase in the proportions of Rorγt + CD4 + TCRβ+ T cells in the lamina propria of SD-IG mice (Fig. 4A, Fig. S4A and Table S4A-D). Recent findings from a murine model of undernutrition and EED induced by malnourished diet and adherent invasive *Escherichia coli* infection likewise reported increased microbiota-directed T regulatory cells, and increased numbers of these cells have also been reported in human cohorts and other murine models of undernutrition [20, 41, 46]. Consistent with these findings, we found increased numbers of Rorγt + FoxP3 + regulatory T cells in the lamina propria of SD-IG mice relative to HD-IG animals (Fig. 4B, S4B). While the majority of innate immune cells were unchanged between groups, we did observe fewer lamina propria macrophages in SD-IG groups (Fig. 4C, S4C), again consistent with data from human cohorts with EED [21]. Interestingly, the lower number of macrophages appeared to be driven by donor 4092 to a greater extent than donor 3114 (0.8 ± 0.3% of live cells for 4092 versus 2.2 ± 1% for donor 3114; Fig. 4C, S4C). Finally, we also noted increased numbers of IgA + Plasma Cells in the lamina propria of SD-IG mice relative to HD-IG mice (Fig. 4D-E, S4D). These cells were also significantly increased in HD-PW mice relative to HD-IG mice. Because the microbial communities of HD mice were similar between IG and PW groups, these results support the idea that the timing of exposure to the microbiota can play a major role in shaping the subsequent host immune response.

### SD-IG mice show elevated CCL5 and IL-1β in the small intestine

To investigate immune signals underlying the observed changes in cell composition in these groups, we next measured changes in small intestinal tissue chemokines and cytokines at the protein level. In ileum tissue lysates, we identified a significant increase in CCL5 (also known as regulated on activation, normal T cell expressed and secreted [RANTES]) protein by ELISA in SD-IG mice relative to HD-IG mice (Fig. 4F, S4E-F). CCL5 production can be induced by the microbiota in other murine models, notably exacerbating inflammation during DSS colitis [47]. *CCL5* gene expression was also upregulated in duodenal biopsies obtained from a cohort of Pakistani patients with EED compared to healthy US controls or patients with celiac disease [19]. Similarly, there was also a trend towards higher Interleukin-1β (IL-1β) in the SD-IG group. IL-1β is an acute phase protein whose secretion is triggered by activation of the inflammasome [47, 48] (Fig. 4G, S4G-H). Overall, levels of these immune signaling molecules were markedly higher in both PW groups relative to both IG groups (Fig. 4G-H).

To further explore potential immune signaling differences between IG and PW colonization, we next performed multiplex bead-based analysis on a subset of ileal tissue lysate samples (Table S5A-B). Of the detectable cytokines and chemokines included in this analysis, we did not identify any additional significant differences between SD and HD groups in either the IG or PW samples. However, several were significantly elevated in both PW groups compared to both IG groups. These included IL-7, IL-10 and IL-17, among others (Table S5A-B). Overall, these results provide further insight into immune outcomes shaped by recognition of the microbiota in early life and support the idea that the timing of exposure to microbial communities plays a critical role in shaping immunity.

## Discussion

Maternal and child undernutrition have increasingly been recognized as intergenerational challenges [4, 49, 50]. Altered gut microbial communities contribute to undernutrition, and infants inherit a significant portion of their microbiota from maternal sources [51]. Despite important observations in human cohorts, murine models of undernutrition that incorporate and investigate intergenerational exposures are lacking. Preclinical models are important in understanding the biological underpinnings and immune consequences of undernutrition in early life. Recent work has shown that targeting the gut microbiota via therapeutic foods can improve child weight gain [52]. This approach was developed based on extensive testing in gnotobiotic animal models, highlighting the important role that preclinical investigation can play in the development of therapies to treat this complex disorder [40]. Indeed, the considerable technical, logistical, and ethical barriers to studying the intergenerational transmission of undernutrition in human populations are well known. Thus, incorporating intergenerational dietary and microbial exposures in a murine model presents a novel opportunity to further clarify the origins of growth stunting driven by undernutrition.

Here we demonstrate that microbiota from two human infant donors with growth stunting can alter linear growth and intestinal villus length, as well as small intestinal immune cell populations relative to microbiota from healthy children. While pooling donors into HD and SD groups allowed us to look for shared phenotypes, differences in the microbial communities between donors also drove variation in certain host phenotypes, including femur length and small intestinal macrophage populations. High levels of inter-individual variation in microbiota composition are frequently observed in human cohorts, and it is thus unsurprising to find that different community compositions impart certain distinct phenotypes [53]. Indeed, this aspect of our model could be used to further identify specific taxa unique to each donor that may drive host phenotypic variation. Our results support the idea that differences in human microbial communities can be informative for understanding specific microbial functions that influence host biology, and highlight the possibility that these differences may influence specific health outcomes in children [54].

Immune and epithelial changes in the small intestine are characteristic of EED and undernutrition, although the functional consequences of these changes are not well understood [16, 20]. Recent work describing intestinal abnormalities in patients with EED at the single cell level showed intriguing similarities to changes identified in this model, including more Granzyme A + TCRγδ + IELs, antibody-producing plasma cells, and fewer intestinal macrophages [21]. Interestingly, increased TCRγδ + IELs were also reported in another murine model of undernutrition and enteropathy [55].While our model was not originally designed to reflect enteropathy, the areas of overlap in immune consequences between these studies suggest this approach may be useful in delineating beneficial or deleterious functions of specific immune populations during undernutrition.

Our results are also broadly consistent with previous work demonstrating that exposure of the immune system to microbial products during the weaning phase is a critical determinant of later life immune function [28, 29]. Germ-free mice are known to have altered immune development that can be partially but not entirely restored after exposure to microbes in adulthood [29, 56]. We observed that differences between animals colonized with microbiota from HD and SD donors were evident when animals are born to colonized parents but are less apparent when animals were directly colonized after weaning. Our results suggest that immune outcomes differ based not only on the presence of a microbiota during early life but also on which microbes are present.

These studies raise questions about the critical time points in early life that shape growth and immunity. While the current body of evidence suggests the weaning phase is one critical time period, many children who develop stunting show lower length-for-age scores at birth, and our results do not rule out a potential role for the maternal gut microbiome during development in utero [11]*.* Furthermore, a weaning reaction to the microbiota has not been demonstrated in humans, so it is unclear how these findings translate to children with undernutrition. However, data from human studies do support an important role for the gut microbiota during early life development [57–59]. Despite these differences, we identified immune alterations in this model (significantly more IELs, regulatory T cells, and plasma cells and fewer macrophages) that have also been shown in patients with undernutrition [19–21], suggesting certain features of the immune response to the microbiota may be shared between mice and humans.

### Limitations of our study

There are several important caveats to the current study. First, due to the complexity and length of the experimental design employed, we were unable to investigate more than four total microbiota donors. While several of our findings show similarities to human studies in patients with undernutrition (significantly more IELs, regulatory T cells and plasma cells and fewer macrophages), the small number of donors employed limits the overall generalizability of our results. We likewise restricted our investigation to microbiota samples from one specific age group (six-month-old infants), and it is unclear how later diversification of the microbiome in children with varied environmental exposures could impact immune composition and function at maturity. Similarly, without having undergone intestinal biopsy, we are unable to determine whether the stunted donors used in this study showed similar intestinal alterations to those observed in gnotobiotic recipients. Additional studies employing intergenerational colonization with an expanded sample size of human donors are necessary to further investigate the link between gut microbial community composition and intestinal physiology, growth, and host immunity in early life. These studies would also benefit from inclusion of pooled samples to reduce donor-to-donor variability, which could improve the consistency of the model for additional mechanistic experimentation.

Another challenge in interpreting our results is the finding that colonization of the two SD microbiota groups differed significantly between PW and IG mice. While interesting, this finding does suggest that observed differences in these groups may be driven by changes in community composition rather than timing of colonization. Likewise, additional testing in this model would be useful to determine whether colonization of dams and sires simultaneously, or dams alone, further shape offspring phenotypes, and could also feature the introduction of undernourished diet at different time points. These studies would open the door to longitudinal characterization of offspring growth, and characterization of the effect of maternal dysbiosis on development in utero. Finally, it also remains to be determined whether this model can recapitulate other long-term sequelae of undernutrition, including oral vaccine failure, increased susceptibility to infectious disease, and cognitive developmental changes. These possibilities likewise warrant further investigation.

## Conclusions

Maternal and child undernutrition are major global health challenges that current therapies do not adequately address. Mechanistic insight into growth and immune pathways regulating development in adverse environments will facilitate the development of targeted therapies to improve global child health. Here we report that intergenerational colonization of gnotobiotic mice with distinct microbial communities leads to altered growth and immune outcomes at maturity. Relative to microbiota from children with healthy growth, microbiota from two donors with linear growth stunting recapitulated several host phenotypes associated with undernutrition (including villous shortening, decreased liver IGF-1, and increased small intestinal IELs and plasma cells). In contrast, colonization of mice after the weaning phase produced fewer phenotypic differences in recipients harboring microbiota from healthy or stunted donors. In summary, we suggest that intergenerational colonization may be a useful approach with which to elucidate the functional role of microbial and immune alterations during undernutrition in early life.

### Supplementary Information

**Supplementary Material 1.**

**Supplementary Material 2.**

**Supplementary Material 3.**

**Supplementary Material 4.**

**Supplementary Material 5.**

**Supplementary Material 6.**

**Supplementary Material 7.**

**Supplementary Material 8.**

## Data Availability

Demultiplexed V4 16 s rRNA sequencing reads have been deposited in the European Nucleotide Archive under accession number PRJEB64154. Sample metadata is provided in Supplementary Table S6. This manuscript does not report original code. All other data needed to evaluate the conclusions in the manuscript are available within the main text or supplementary materials.
