# Supplementary figures and images for: Colonization during a key developmental window reveals microbiota-dependent shifts in growth and immunity during undernutrition
Source: Microbiome. 2024 Apr 9;12:71. doi: 10.1186/s40168-024-01783-3 (PMC11003143; doi:10.1186/s40168-024-01783-3)

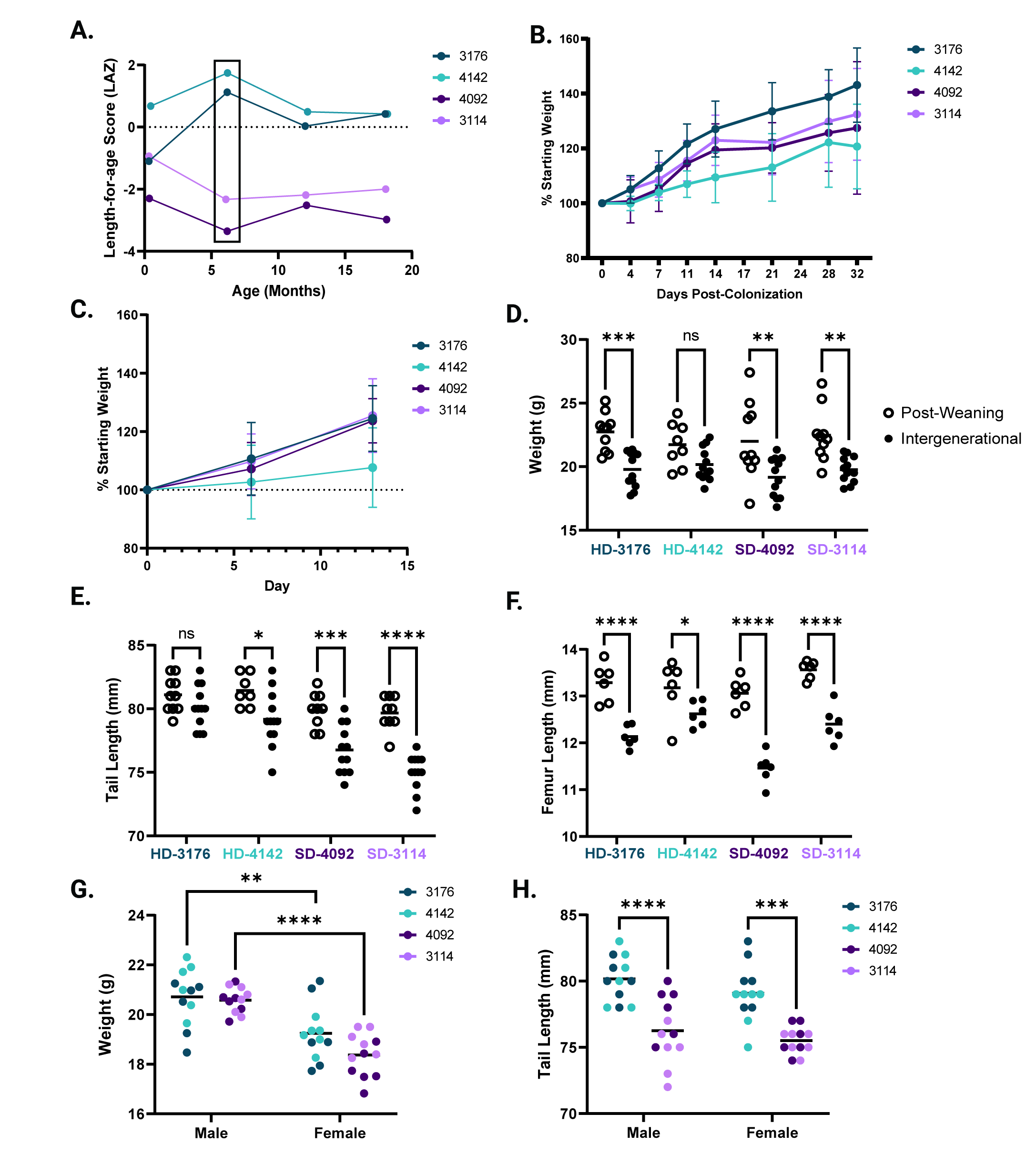

Supplement: Supplementary file 3 — Supplementary Material 2. [file 40168_2024_1783_MOESM2_ESM.tif]

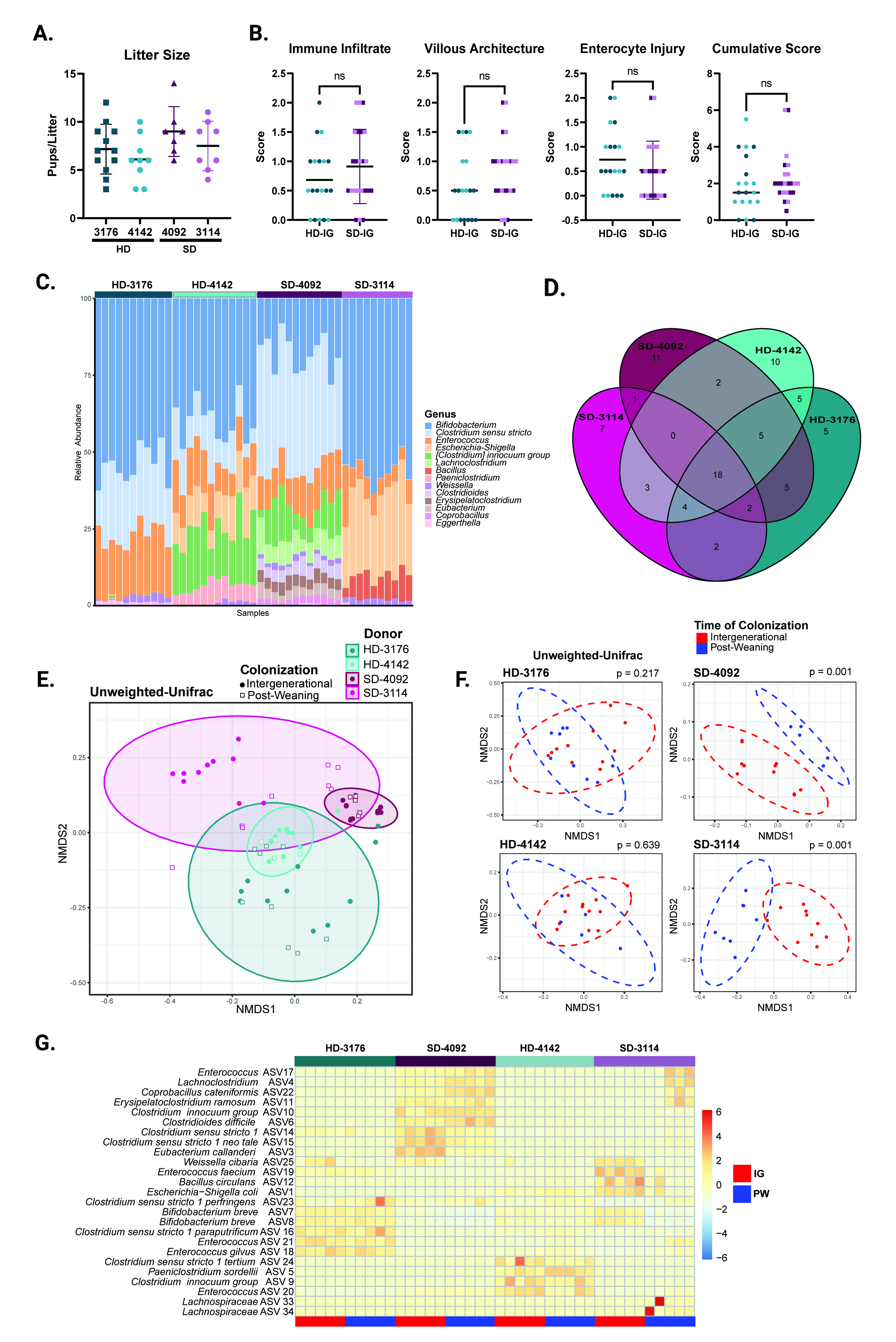

Supplement: Supplementary file 4 — Supplementary Material 3. [file 40168_2024_1783_MOESM3_ESM.tif]

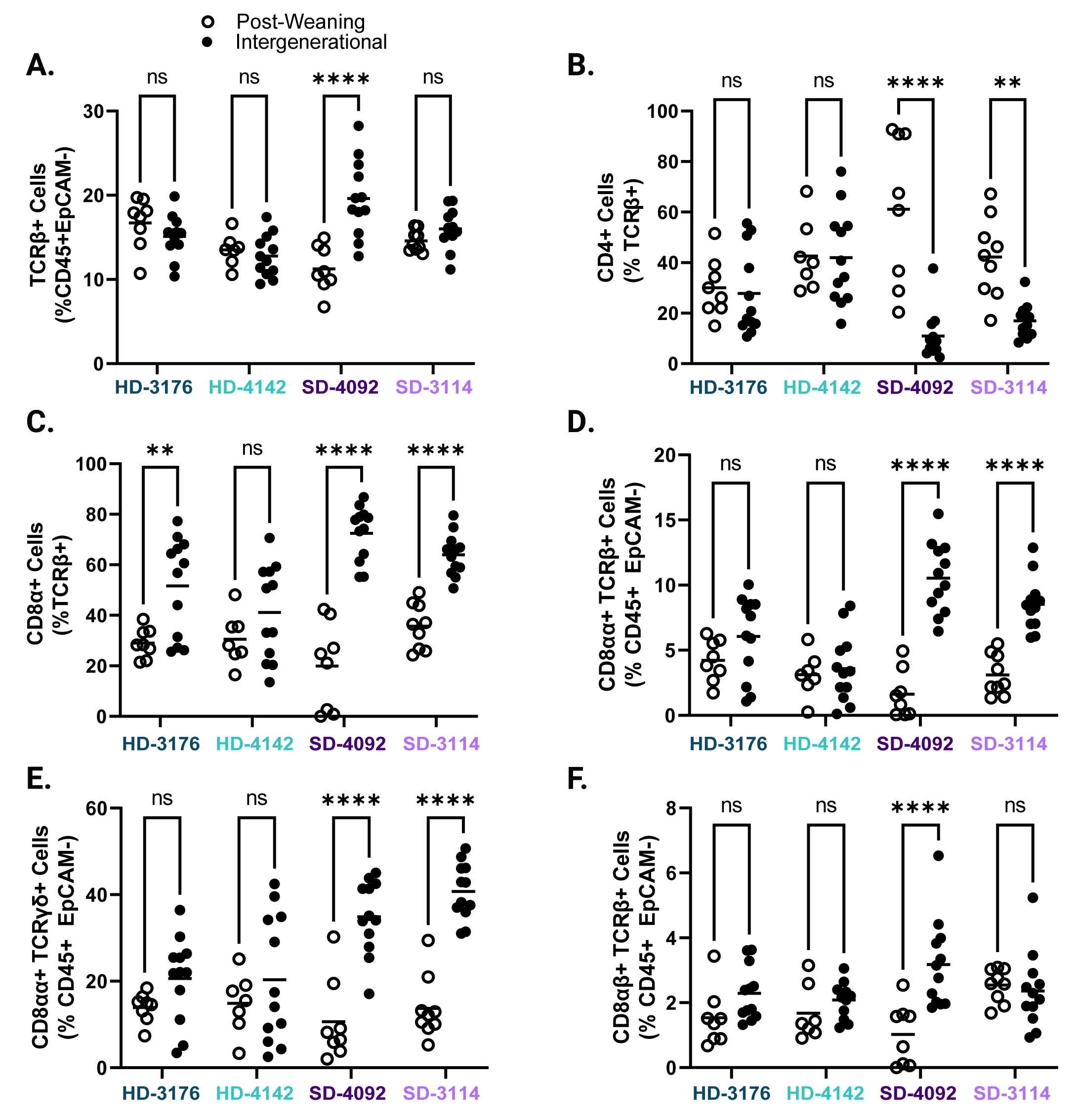

Supplement: Supplementary file 5 — Supplementary Material 4. [file 40168_2024_1783_MOESM4_ESM.tif]

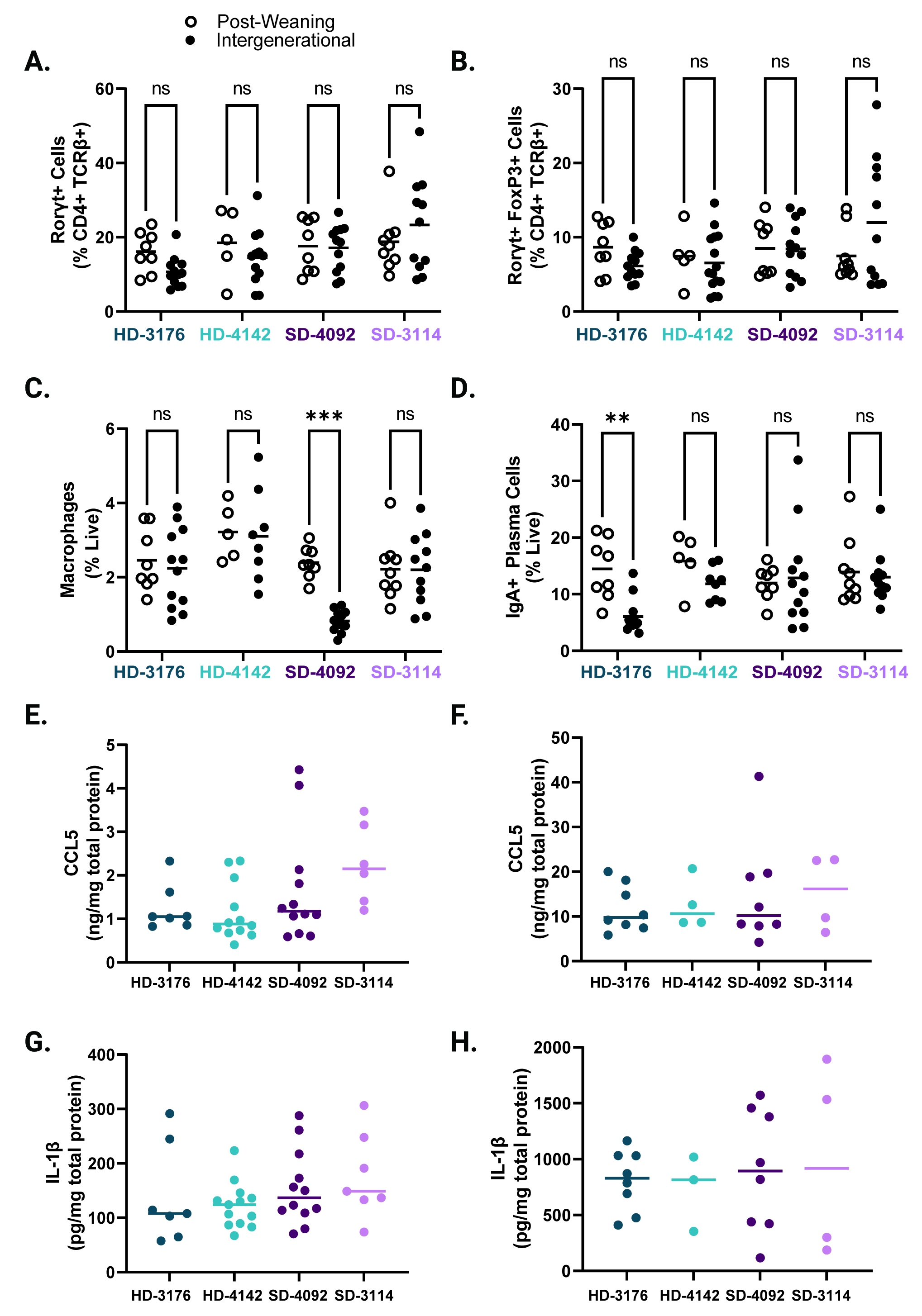

Supplement: Supplementary file 6 — Supplementary Material 5. [file 40168_2024_1783_MOESM5_ESM.tif]

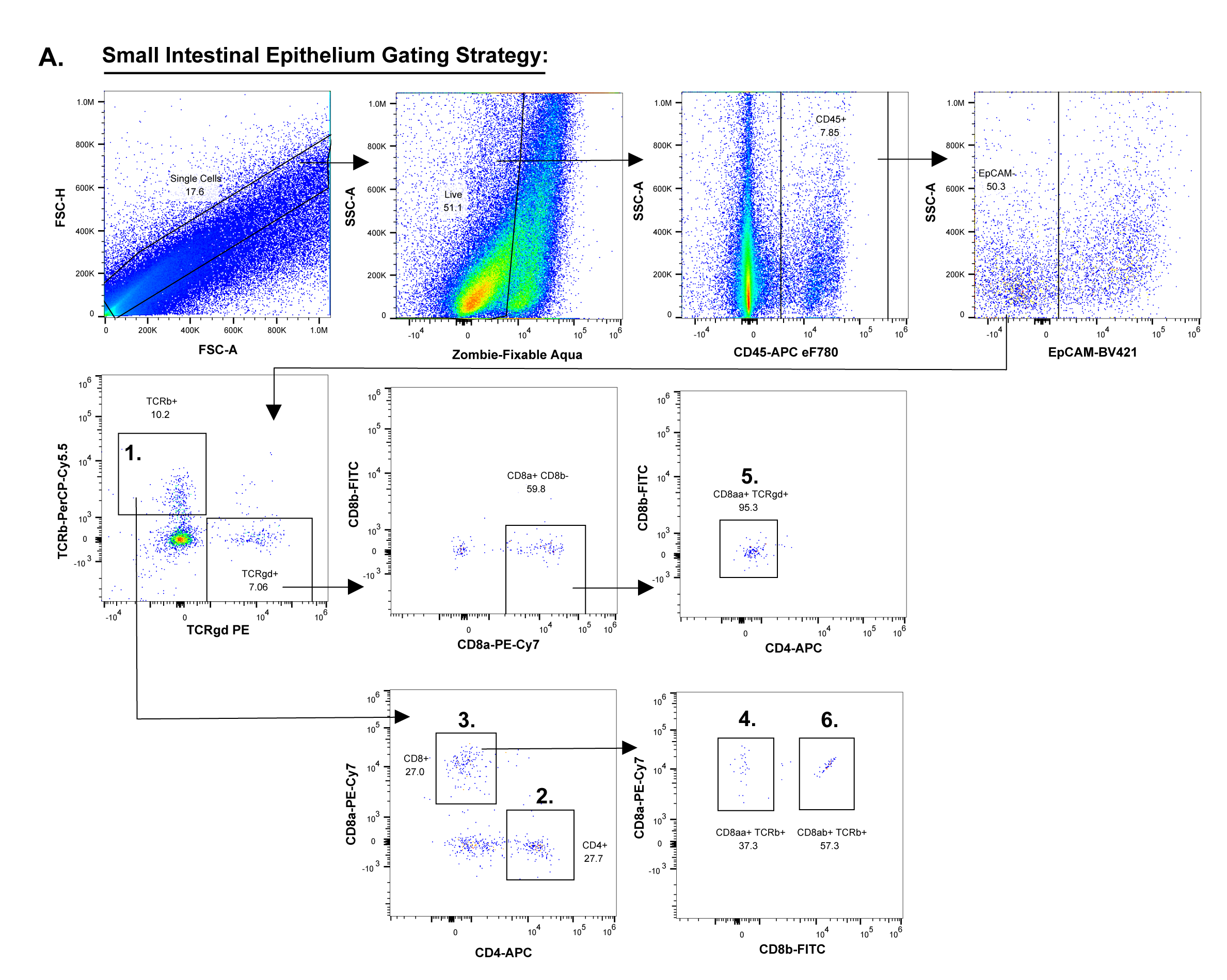

Supplement: Supplementary file 7 — Supplementary Material 6. [file 40168_2024_1783_MOESM6_ESM.tif]

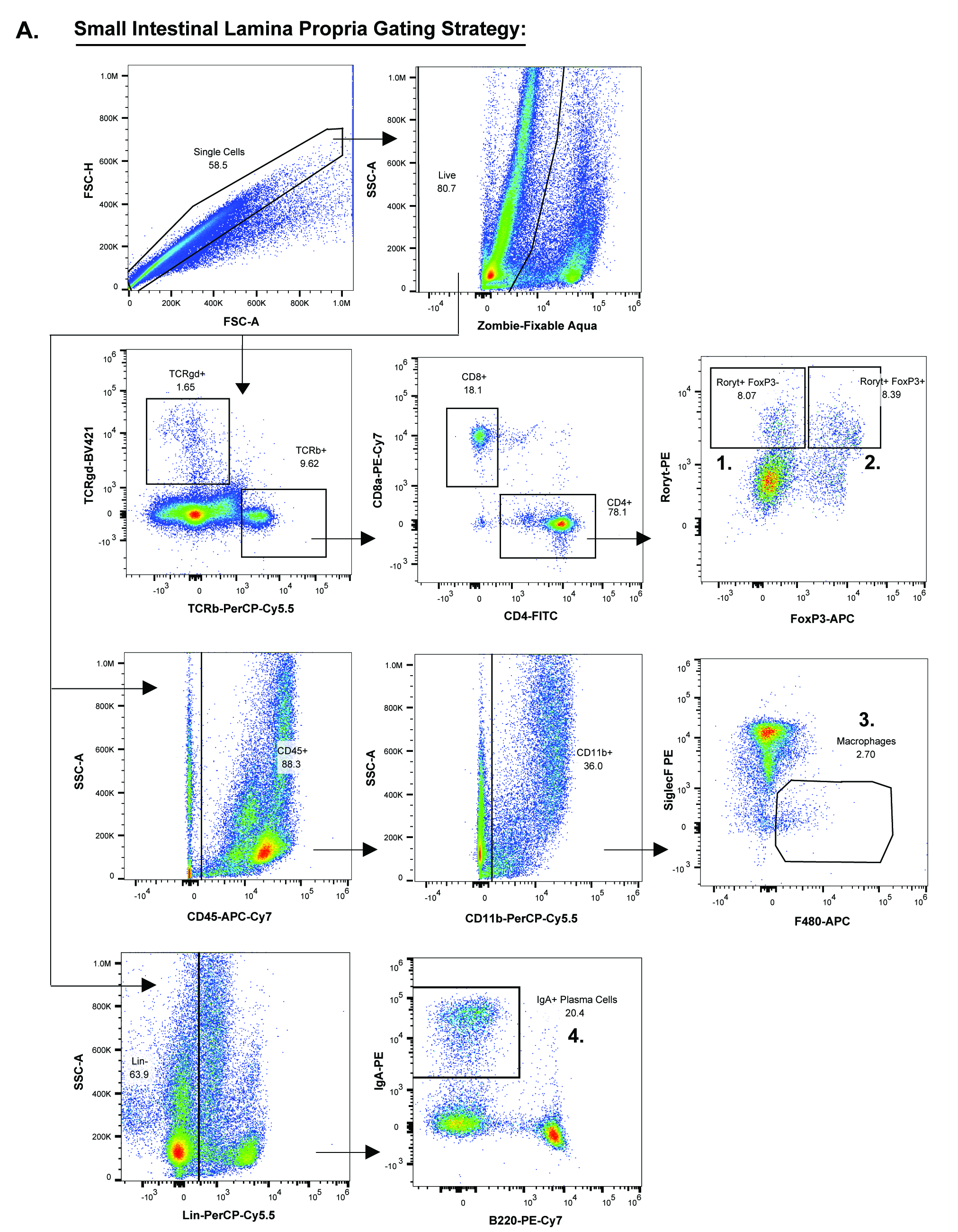

Supplement: Supplementary file 8 — Supplementary Material 7. [file 40168_2024_1783_MOESM7_ESM.tif]

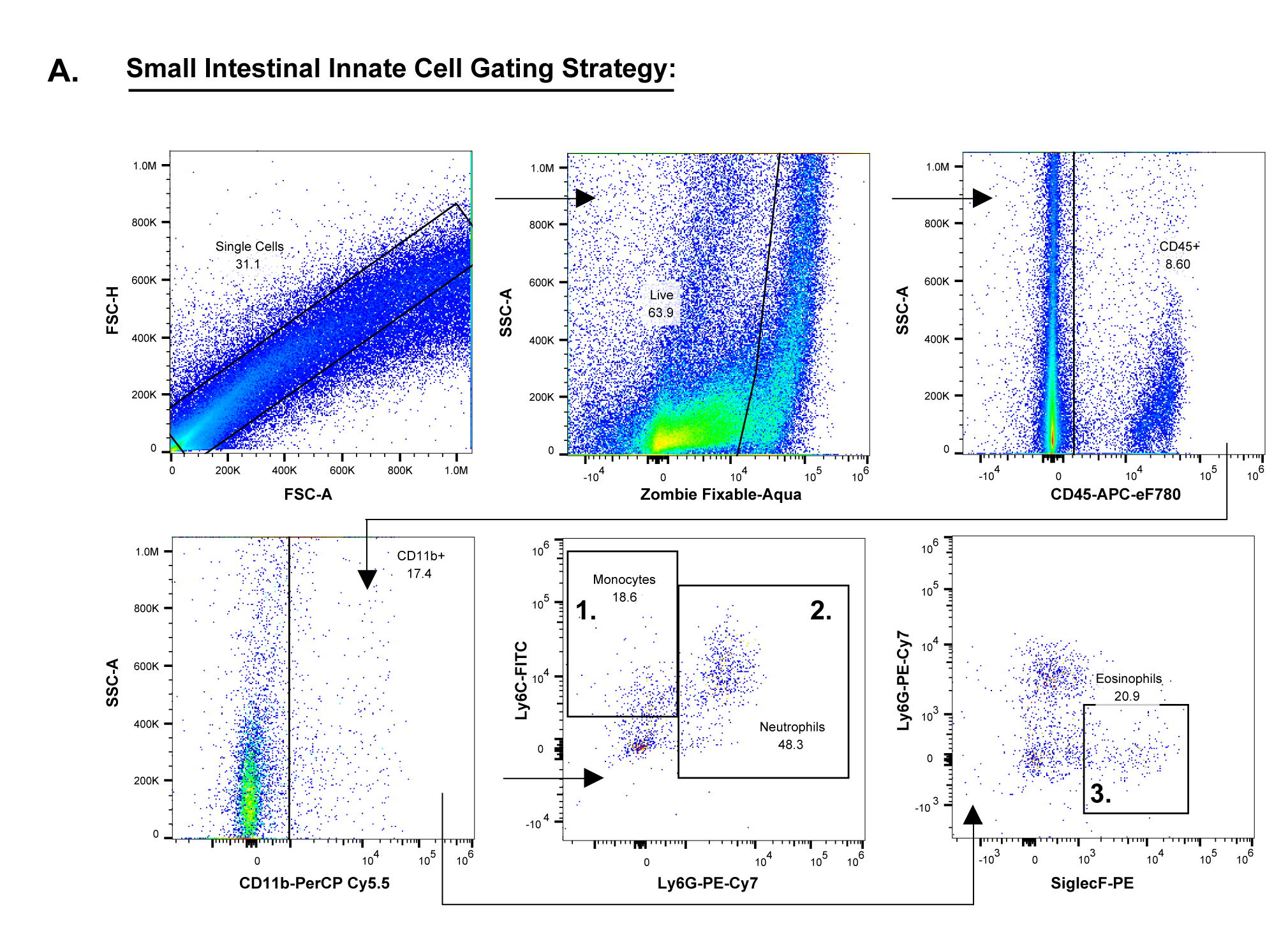

Supplement: Supplementary file 9 — Supplementary Material 8. [file 40168_2024_1783_MOESM8_ESM.tif]
